# Cytomegalovirus Viremia as a Risk Factor for Mortality Prior to Antiretroviral Therapy among HIV-Infected Gold Miners in South Africa

**DOI:** 10.1371/journal.pone.0025571

**Published:** 2011-10-12

**Authors:** Katherine Fielding, Ai Koba, Alison D. Grant, Salome Charalambous, John Day, Cedric Spak, Anna Wald, Meei-Li Huang, Lawrence Corey, Gavin J. Churchyard

**Affiliations:** 1 Department of Infectious Disease Epidemiology, London School of Hygiene and Tropical Medicine, London, United Kingdom; 2 Department of Clinical Research, London School of Hygiene and Tropical Medicine, London, United Kingdom; 3 The Aurum Institute, Johannesburg, South Africa; 4 Department of Medicine, University of Washington, Seattle, Washington, United States of America; 5 Department of Laboratory Medicine, University of Washington, Seattle, Washington, United States of America; 6 Department of Epidemiology, University of Washington, Seattle, Washington, United States of America; 7 Vaccine and Infectious Diseases Division, Fred Hutchinson Cancer Research Center, Seattle, Washington, United States of America; Institut National de la Santé et de la Recherche Médicale, France

## Abstract

**Background:**

Cytomegalovirus (CMV) viremia has been shown to be an independent risk factor for increased mortality among HIV-infected individuals in the developing world. While CMV infection is nearly ubiquitous in resource-poor settings, few data are available on the role of subclinical CMV reactivation on HIV.

**Methods:**

Using a cohort of mineworkers with stored plasma samples, we investigated the association between CMV DNA concentration and mortality prior to antiretroviral therapy availability.

**Results:**

Among 1341 individuals (median CD4 count 345 cells/µl, 70% WHO stage 1 or 2, median follow-up 0.9 years), 70 (5.2%) had CMV viremia at baseline; 71 deaths occurred. In univariable analysis CMV viremia at baseline was associated with a three-fold increase in mortality (hazard ratio [HR] 3.37; 95% confidence intervals [CI] 1.60, 7.10). After adjustment for CD4 count, WHO stage and HIV viral load (N = 429 with complete data), the association was attenuated (HR 2.27; 95%CI 0.88, 5.83). Mortality increased with higher CMV viremia (≥1,000 copies/ml vs. no viremia, adjusted HR 3.65, 95%CI: 1.29, 10.41). Results were similar using time-updated CMV viremia.

**Conclusions:**

High copy number, subclinical CMV viremia was an independent risk factor for mortality among male HIV-infected adults in South Africa with relatively early HIV disease. Studies to determine whether anti-CMV therapy to mitigate high copy number viremia would increase lifespan are warranted.

## Introduction

Cytomegalovirus (CMV) is a major opportunistic pathogen among HIV-infected persons. Not only is it important because of its direct pathogenicity leading to CMV end-organ disease and death in severely immunosuppressed individuals, typically those with CD4 cell counts of less than 100 cells/µl[1], [2], but also because of its potential role in HIV disease progression [3], [4].

Since the 1980s, a number of studies investigated the association between CMV and mortality among HIV-infected individuals. However, most early studies used CMV antibody positivity as the exposure of interest[5], [6], [7]. Quantification of CMV viremia is a more precise marker of CMV activity and more recent studies have used this as the exposure of interest[8], [9], [10], [11], [12], [13], but many of these have been in cohorts receiving antiretroviral therapy (ART), among whom it is more difficult to tease out the effect of CMV on natural history of HIV infection. Most persons who are CMV antibody positive harbour latent rather than active CMV infection. As such, virological markers of ongoing CMV replication are likely to be more accurate predictors of the association between CMV reactivation and HIV mortality. Subclinical CMV infection is a well known risk factor for mortality in organ and bone marrow transplant recipients[14], [15].

Between 1999 and 2002 we conducted a cohort study[16] among employees of a gold mine in South Africa attending a newly-established HIV clinic, prior to the availability of ART; as part of this study, serial blood samples were stored. This created an opportunity to investigate the effect of CMV viremia on mortality in this cohort.

## Methods

### Ethical statement

The study was approved by Research Ethics Committees of the University of KwaZulu Natal, South Africa; the London School of Hygiene and Tropical Medicine, UK; and the Institutional Review Board of the Fred Hutchinson Cancer Research Center, Seattle, USA.

### Study design, population and data collection

This analysis used data from a workplace HIV clinic within a large gold mining company in the Free State Province, South Africa, in the context of a prospective cohort study (the "parent study") to explore the effect of tuberculosis on HIV viral load between May 1999 and March 2002[16]. The clinic was administered by the company and provided employees with free, comprehensive health care. In 1999, specialized care for HIV-infected employees was added[17]. Voluntary counselling and testing for HIV infection was encouraged for persons with a sexually transmitted infection, or tuberculosis; persons testing HIV positive were invited to the clinic. All patients were screened for active tuberculosis at clinic entry and offered isoniazid preventive therapy if they had no evidence of active tuberculosis or history of prior tuberculosis treatment, and cotrimoxazole preventive therapy if the CD4 count was below 200 cells/µl, or if they had symptomatic HIV disease and a CD4 count below 250 cells/µl. Patients were seen for routine follow up every six months. As part of routine clinic procedures, at every visit, data were collected on demographic and medical history, physical examination, World Health Organization (WHO) disease stage, and clinical laboratory analyses including measurement of CD4 cell count. Neither ART nor quantification of HIV load was routinely available at the time of the study.

Clinic patients who were 18 years of age or older were invited to enrol in the parent study, regardless of CD4 cell count, and for those consenting, plasma samples obtained at routine visits were stored. All participants in the parent study who had stored plasma available for testing for CMV and who had a CD4 count available within ± six months of their first (defined as “baseline”) stored plasma sample were eligible for this analysis. Mortality information was obtained from medical and employment records. Because CMV infection is ubiquitous amongst adults in developing countries[18], we did not test for CMV antibodies. Stored plasma from all samples available for each participant were analysed retrospectively at the University of Washington for CMV DNA in a previously validated assay. HIV RNA concentration was measured at baseline on all subjects with CMV viremia and on a randomly selected subset of participants without CMV viremia, using the same plasma sample as the baseline CMV measurement.

### Laboratory methods

DNA was extracted from 200 ul of stored plasma and eluted into 100 ul of AE buffer (Qiagen Inc). Ten ul of DNA was then analyzed by CMV specific TaqMan real-time PCR as previously described[19]. The lower limit of detection of CMV DNA was 50 copies/ml. HIV-1 particles were pelleted by centrifugation from 500 µl plasma. RNA was extracted from the pellet and resuspended in 50 ul of AE buffer. Twenty ul of RNA was then used to detect HIV RNA by real-time RT- PCR as described previously[20].

### Statistical analysis

The chi-square test or Fisher's exact test, and Wilcoxon rank sum test were used to assess the association of categorical and quantitative variables, respectively, with baseline CMV viremia.

The primary outcome for this analysis was death. Participants were considered to enter the cohort on the date of the first available CMV measurement (baseline), and were followed until death, leaving the work force, or end of study (31^st^ March 2002), whichever came first. CD4 count and WHO stage at baseline were defined as the closest measurement, within ± six months, to the date of the baseline CMV measurement.

Cox proportional hazards regression was used to estimate the effect of baseline CMV viremia on time to death. Data were reported as hazard ratios (HRs), 95% confidence intervals (CIs), and P values from the likelihood ratio test (LRT). CMV viremia status was defined as positive (>50 copies/ml) or negative and as a variable on three levels (negative, 51–999, ≥1000 copies/ml). Where appropriate, tests for linear trend and departures from linear trend were assessed using the LRT. Non-proportional hazards for CMV and other factors were explored. A multivariable analysis was conducted to assess the relationship between CMV viremia and death, controlling for potential confounders including age, CD4 cell count and WHO stage. An interaction between CD4 cell count (<200 versus ≥200 cells/µl) and CMV viremia was defined *a priori* and was assessed using the LRT. The analysis was repeated on a subset of individuals with data on HIV RNA concentration. Time to death by CMV viremia status measured at baseline was summarized graphically by Kaplan-Meier survival curves, stratified by CD4 count (<200 and ≥200 cells/µl) at baseline.

A sensitivity analysis was conducted using all available CMV measurements during follow-up, therefore modelling CMV viremia as a time-updated covariate.

Data were analysed using STATA v. 10 (Stata Corporation, College Station, Texas, USA).

## Results

### Baseline characteristics and CMV status

Between May 1999 and March 2002, 1,342 patients were enrolled in the parent study, and had suitable stored specimens available for analysis for CMV viremia. One patient had missing baseline WHO stage, and so analysis was restricted to 1,341 patients. The population was predominantly male (1,335; 99.6%) with a median age of 39 (range 19–59) years, consistent with the demographics of the gold mining workforce (table 1). The median baseline CD4 cell count was 345 cells/µl (interquartile range [IQR]: 218–508), 29% had a previous episode of TB and 70% (934) were in WHO stage 1 or 2 (table 1). None were receiving ART.

**Table 1:** Description of cohort overall, and stratified by CMV viremia at baseline.

		All participants	CMV viremia: >50 copies/ml a	Negative for CMV viremia	P value^b^
		(n = 1341)	(n = 70)	(n = 1271)	
Age (years)	*Median (IQR)*	39 (35–45)	40 (36–46)	39 (35–44)	0.33
CD4 count (cells/µL)	*Median (IQR)*	345 (218, 508)	213 (108–318)	354 (225–517)	<0.0001

		*n*	*(col %)*	*n*	*(col %)*	*n*	*(col %)*	
Sex	Male	1335	(99.6)	70	(100)	1265	(99.5)	>0.99
CD4 count (cells/µL)	<100	83	(6.2)	16	(22.9)	67	(5.3)	<0.0001
	100–199	196	(14.6)	15	(21.4)	181	(14.2)	
	200–349	401	(29.9)	26	(37.1)	375	(29.5)	
	≥350	661	(49.3)	13	(18.6)	648	(51.0)	
WHO stage	1 or 2	934	(69.6)	41	(58.6)	893	(70.3)	0.001
	3	328	(24.5)	18	(25.7)	310	(24.4)	
	4	79	(5.9)	11	(15.7)	68	(5.4)	
HIV viral load^c^ (log_10_ copies/ml)	<4	139	(32.4)	9	(12.9)	130	(36.2)	<0.0001
	4–5	158	(36.8)	28	(40.0)	130	(36.2)	
	≥5	132	(30.8)	33	(47.1)	99	(27.6)	
Previous tuberculosis	Yes	393	(29.3)	30	(42.9)	363	(28.6)	0.01

a n = 42 and n = 28 have CMV DNA levels of between 51–999 and ≥1000 copies/ml, respectively;

b comparing CMV viremia versus no viremia at baseline using chi-square test or Fisher's exact test for categorical variables and the Wilcoxon rank sum test for quantitative variables;

c n = 429 with HIV viral load data

IQR: interquartile range; col = column; WHO stage = World Health Organization HIV clinical stage.

The median number of CMV measurements per patient, including the baseline sample, was two (range one to nine) with a total of 2703 measurements overall. Including all measurements, 146 participants (10.9%) had at least one sample with detectable CMV viremia. Of the 770 participants with at least two measurements, 84.3% (649) were consistently negative (median of two measurements), 1.4% (11) consistently positive (median of two measurements) and 14.3% (110) had their CMV viremia status change at least once during follow-up (median of three measurements). The median time between the first and second measurement was 6.6 months. Of participants with a baseline CD4 count≥200 cells/µl, by 6 months 89.5% (453/506) maintained a CD4≥200, with a median decrease of 26 cells/µl (IQR 26–86) over that period.

Of the 1,341 patients, 70 (5.2%) had detectable CMV viremia at baseline with a median CMV DNA level of 524 copies/ml (IQR: 116–1994) and 40% of those had CMV DNA level ≥1000 copies/ml. Those with detectable CMV viremia had a lower median CD4 cell count (213 vs. 354 cells/µl, p<0.0001), higher HIV RNA concentration (47.1% vs. 27.6% with ≥5log_10_ copies/ml, p<0.0001) and a higher proportion were in WHO stage 3 or 4 (43% vs. 30%, p = 0.001), compared to those without CMV viremia (table 1).

### Association of baseline CMV viremia and time-updated CMV viremia with mortality

**Figure 1:**
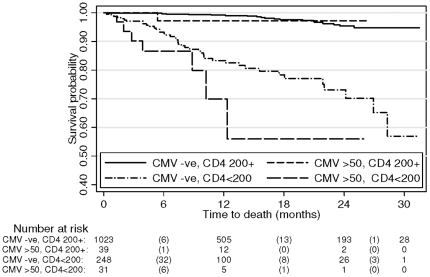
Kaplan-Meier curves by CMV viremia and CD4 count at baseline. Footnote to figure 1: The total number of participants at risk at 0, 12, 24 and 30 months of follow-up and the total number of deaths (in parentheses) during the intervals 0–12, 12–24 and 24–30 months, by CMV viremia (-ve, >50 copies/ml) and CD4 count (<200, ≥200 cells/µL), are shown.

The median follow-up time was 0.9 years (range 0.01–2.3) with a total of 1,534 person years of observation, during which 71 deaths occurred. In unadjusted analysis, CMV viremia at baseline was associated with a greater than three-fold increased hazard of death (unadjusted HR 3.37; 95% CI 1.60, 7.10; P = 0.006, table 2). After adjusting for CD4 cell count and WHO stage, the HR was substantially attenuated to 1.17 (95% CI 0.52, 2.65; P = 0.71). Kaplan-Meier survival curves for CMV viremia at baseline, stratified by CD4 count (<200 and ≥200 cells/µl) are shown in figure 1. Age did not confound the association, and was therefore not included in the model. There was no evidence for an interaction between CD4 group (<200, ≥200 cells/µL) and CMV viremia (P = 0.87). In the unadjusted analysis there was a trend for increasing mortality risk with increasing levels of CMV viremia (P for trend 0.002), though after adjustment only those with ≥1000 copies/ml had evidence of an increased hazard of death (adjusted HR 2.02, 95% CI 0.78–5.27) compared to those without CMV viremia.

**Table 2:** Effect baseline and time-updated CMV viremia on mortality, unadjusted and adjusted hazard ratios from Cox proportional hazards regression, 95% confidence intervals and P values (n = 1341).

		Deaths/ pyrs	Rate/ 100 pyrs	Unadjusted HR	Model a: Adjusted HR^a^ (95% CI)	Model b: Adjusted HR^a^ (95% CI)	Model c: Adjusted HR^a^ (95% CI)	Model d: Adjusted HR^a^ (95% CI)
					*(Baseline CMV viremia)*		*(Time-updated CMV viremia)*	
CMV viremia at baseline (copies/ml)	-ve	63/1475	4.3	1 (p = 0.006)	1 (p = 0.71)	N/A	N/A	N/A
	>50	8/59	13.5	3.37	1.17 (0.52–2.65)			
CMV viremia at baseline (copies/ml)	-ve	63/1475	4.4	1 (p = 0.006^b^)	N/A	1 (p = 0.28)	N/A	N/A
	51-999	3/38	7.8	1.96		0.65 (0.19-2.24)		
	≥1000	5/21	23.9	5.99		2.02 (0.78–5.27)		
CMV viremia time-updated (copies/ml)	-ve	59/1451	4.1	1 (p<0.001)	N/A	N/A	1 (p = 0.53)	N/A
	>50	12/83	14.4	3.52			1.25 (0.63–2.47)	
CMV viremia time-updated (copies/ml)	-ve	59/1451	4.1	1 (p<0.001^c^)	N/A	N/A	N/A	1 (p = 0.009)
	51–999	3/55	5.5	1.30				0.45 (0.14–1.49)
	≥1000	9/28	31.9	8.13				2.85 (1.35, 6.02)
CD4 count d (cells/µL)	<100	26/60	43.2	81.4	64.1 (23.5–175)	64.8 (23.8–176)	62.6 (22.9–171)	64.7 (23.6–177)
	100–199	24/211	11.4	18.3	16.4 (6.23–43.4)	15.9 (6.00–42.0)	16.4 (6.19–43.2)	16.1 (6.10–42.6)
	200–349	16/481	3.3	5.19	4.88 (1.78–13.4)	4.90 (1.79–13.4)	4.85 (1.77–13.3)	4.78 (1.75–13.1)
	≥350	5/782	0.6	1 (p<0.001)	1 (p<0.001)	1 (p<0.001)	1 (p<0.001)	1 (p<0.001)
								
WHO stage	1–2	32/1121	2.8	1 (p<0.001)	1 (p = 0.16)	1 (p = 0.13)	1 (p = 0.18)	1 (p = 0.17)
	3	29/340	8.5	3.04	1.62 (0.96–2.73)	1.65 (0.98–2.78)	1.61 (0.96–2.71)	1.61 (0.96–2.71)
	4	10/73	13.7	5.08	1.58 (0.72–3.49)	1.70 (0.78–3.71)	1.54 (0.71–3.38)	1.57 (0.72–3.46)
Age (years)	<35	9/394	2.3	1 (p = 0.1)	Not adjusted for	Not adjusted for	Not adjusted for	Not adjusted for
	35–39	26/436	6.0	2.62				
	40–44	18/353	5.1	2.23				
	45–49	13/246	5.3	2.43				
	≥50	5/105	4.8	2.14				

a adjusted for all variables shown; P value shown in parentheses;

b P-value for trend = 0.002;

c P-value for trend<0.001;

d CD4 categories are <100, 100-199, 200-349 and ≥350 cells/µL.

pyrs = person years; HR = hazard ratio; CI = confidence interval; -ve = negative; N/A = not applicable; WHO stage = World Health Organization HIV clinical stage.

Results were similar for the sensitivity analysis when CMV viremia was time-updated using all available CMV viremia measurements in follow-up (table 2). The unadjusted HR for mortality comparing those with versus without CMV viremia was 3.52 (95% CI 1.89, 6.55; P<0.001) and after adjustment for baseline CD4 cell count and WHO stage the HR was 1.25 (95% CI 0.63, 2.47; P = 0.53). However, for those with CMV viremia ≥1000 copies/ml versus those without CMV viremia, the unadjusted and adjusted HRs were 8.13 (95% CI 4.02, 16.32) and 2.85 (95% CI 1.35, 6.02), respectively, indicating that the development of high copy number CMV plasma viremia was an independent risk factor for increased mortality.

Analysis restricted to 429 patients with HIV RNA concentration data (27 deaths) showed consistent evidence of an increased hazard of death for those with CMV viremia at baseline after adjustment for CD4 cell count, WHO stage and HIV viral load (adjusted HR 2.27, 95% CI 0.88, 5.83, p = 0.11). There was again no evidence for an interaction between CD4 group (<200, ≥200 cells/µl) and CMV viremia defined as positive or negative (P = 0.71). For CMV viremia defined on three levels, after adjustment, increased mortality was observed with with increasing levels of CMV viremia (P for trend 0.04); those with ≥1000 copies/ml had an adjusted HR of 3.65 (95% CI 1.29, 10.41) compared to being CMV negative. Results were similar for the analysis based on time-updated CMV viremia (data not shown).

## Discussion

This retrospective laboratory evaluation of a prospective cohort study of HIV-infected sub-Saharan African men found a consistent association between CMV viremia, especially high copy levels of CMV DNA in plasma, and increased hazard of death. These data are unusual in that they come from a population of miners in South Africa, many of whom had not reached the stage of advanced HIV disease, and in this context suggest that the association between CMV and mortality is not restricted to individuals with advanced HIV-related disease or those with overt CMV end-organ damage.

The prevalence at baseline of CMV viremia in our study, at 5%, was somewhat lower than has been reported in other studies[10], [12], [21], [22], despite using an assay which could detect CMV viremia at concentrations of 50 copies/ml. Whether this is a true biological phenomenon, or the difference between serum and plasma will require more detailed evaluation. Overall, 11% of patients had at least one specimen with detectable CMV viremia. This is consistent with the relatively high median CD4 count among our participants. Our data contribute to the very limited information concerning the prevalence of CMV viremia among people with HIV infection in resource-limited settings. In a small study of pregnant women in Kenya, 17% had CMV viremia; the median CD4 count was 335 cells/µl among those with CMV viremia and 420 cells/µl among those without[21]; post-partum survival was shorter among women with CMV viremia. Among 377 adults newly-diagnosed with HIV infection in Cambodia, 55.2% had CMV viremia; this very high prevalence may be explained by the group being severely immunosuppressed with a median CD4 count of 30 cells/µl[22].

Studies in the U.S. and Europe among patients on ART have also found associations between CMV viremia and increased risk of death, with hazard ratios ranging from nearly 2 to almost 5 after controlling for other indicators of disease progression such as CD4 cell count and HIV viral load[10], [11], [12], [13]. Some studies have reported that the risk of death has a linear association with the log_10_ CMV DNA concentration. Bowen *et al*. and Spector et al. reported that each log_10_ increase in CMV DNA load was associated with a 1.9-fold and 2.2-fold increase in mortality, respectively[12], [13]. Among adults in Cambodia, Micol *et al*. found that CMV viral load greater than 3.1log_10_ copies/ml was independently associated with death[22]. To our knowledge, this is the first study of persons in sub-Saharan Africa with relatively high CD4 counts showing an independent association between high copy numbers of CMV viremia and subsequent mortality.

Assessment of causality is difficult in studies such as ours, and thus we cannot determine whether higher CMV viremia directly increases the risk of death, or whether it is a marker of higher mortality risk attributable, for example, to impaired immune function, independent of the CD4 count. However a randomised trial of CMV-specific therapy would be needed to address this question, most likely in addition to antiretroviral therapy. A cohort study conducted in the US, found that treatment with systemic anti-CMV treatment was associated with a 28% reduction in mortality amongst patients with CMV retinitis, independent of HAART and using time-updated covariates [23]. Overall, however, data documenting the cofactor effect of CMV on HIV disease progression are accumulating[3]. In addition, the deleterious effect of CMV viremia in persons with a higher CD4 count, who are not candidates for antiretroviral therapy according to current guidelines for resource-poor countries, suggests that CMV diagnostics and therapeutics should be considered for evaluation in these settings.

Our study has some limitations. First, although we had a large cohort of 1,342 participants, only a subset had data on HIV RNA. In the multivariable analysis, the association between CMV and mortality was stronger after adjusting for HIV RNA load, suggesting negative confounding. This observation is consistent with the hypothesis that the mechanism of action of CMV on risk of death is independent of its action on plasma HIV RNA[3]. Second, our cohort was not treated with antiretroviral therapy, limiting the ability to generalize the findings to patients receiving current standard of care treatment. Since 2003 goldminers have had access to ART, free-of-charge. Earlier initiation of ART, as per current guidelines in South Africa, is likely to be beneficial in reducing mortality, including that related to CMV. Anti-CMV interventions for those with higher CD4 counts could be worth exploring.

In summary, we found associations between CMV viremia and increased risk of death in a population of gold miners with HIV infection, but mostly without advanced immunosuppression, prior to the ART era. Earlier initiation of ART, as per current guidelines in South Africa, may be helpful in reducing mortality. Further research on interventions to prevent high copy number subclinical CMV viremia may be warranted.

## References

[1] Drew WL (1992). Cytomegalovirus infection in patients with AIDS.. Clin Infect Dis.

[2] Gallant JE, Moore RD, Richman DD, Keruly J, Chaisson RE (1992). Incidence and natural history of cytomegalovirus disease in patients with advanced human immunodeficiency virus disease treated with zidovudine. The Zidovudine Epidemiology Study Group.. J Infect Dis.

[3] Griffiths PD (2006). CMV as a cofactor enhancing progression of AIDS.. J Clin Virol.

[4] Webster A, Grundy JE, Lee CA, Emery VC, Cook DG (1989). Cytomegalovirus infection and progression to AIDS.. Lancet.

[5] Robain M, Boufassa F, Hubert JB, Persoz A, Burgard M (2001). Cytomegalovirus seroconversion as a cofactor for progression to AIDS.. AIDS.

[6] Kovacs A, Schluchter M, Easley K, Demmler G, Shearer W (1999). Cytomegalovirus infection and HIV-1 disease progression in infants born to HIV-1-infected women. Pediatric Pulmonary and Cardiovascular Complications of Vertically Transmitted HIV Infection Study Group.. N Engl J Med.

[7] Sabin CA, Phillips AN, Lee CA, Janossy G, Emery V (1995). The effect of CMV infection on progression of human immunodeficiency virus disease is a cohort of haemophilic men followed for up to 13 years from seroconversion.. Epidemiol Infect.

[8] Jabs DA, Holbrook JT, Van Natta ML, Clark R, Jacobson MA (2005). Risk factors for mortality in patients with AIDS in the era of highly active antiretroviral therapy.. Ophthalmology.

[9] Wohl DA, Zeng D, Stewart P, Glomb N, Alcorn T (2005). Cytomegalovirus viremia, mortality, and end-organ disease among patients with AIDS receiving potent antiretroviral therapies.. J Acquir Immune Defic Syndr.

[10] Deayton JR, Prof Sabin CA, Johnson MA, Emery VC, Wilson P (2004). Importance of cytomegalovirus viraemia in risk of disease progression and death in HIV-infected patients receiving highly active antiretroviral therapy.. Lancet.

[11] Erice A, Tierney C, Hirsch M, Caliendo AM, Weinberg A (2003). Cytomegalovirus (CMV) and human immunodeficiency virus (HIV) burden, CMV end-organ disease, and survival in subjects with advanced HIV infection (AIDS Clinical Trials Group Protocol 360).. Clin Infect Dis.

[12] Spector SA, Wong R, Hsia K, Pilcher M, Stempien MJ (1998). Plasma cytomegalovirus (CMV) DNA load predicts CMV disease and survival in AIDS patients.. J Clin Invest.

[13] Bowen EF, Wilson P, Cope A, Sabin C, Griffiths P (1996). Cytomegalovirus retinitis in AIDS patients: influence of cytomegaloviral load on response to ganciclovir, time to recurrence and survival.. AIDS.

[14] Burak KW, Kremers WK, Batts KP, Wiesner RH, Rosen CB (2002). Impact of cytomegalovirus infection, year of transplantation, and donor age on outcomes after liver transplantation for hepatitis C.. Liver Transpl.

[15] George B, Pati N, Gilroy N, Ratnamohan M, Huang G (2010). Pre-transplant cytomegalovirus (CMV) serostatus remains the most important determinant of CMV reactivation after allogeneic hematopoietic stem cell transplantation in the era of surveillance and preemptive therapy.. Transpl Infect Dis.

[16] Day JH, Grant AD, Fielding KL, Morris L, Moloi V (2004). Does tuberculosis increase HIV load?. J Infect Dis.

[17] Charalambous S, Grant AD, Day JH, Rothwell E, Chaisson RE (2004). Feasibility and acceptability of a specialist clinical service for HIV-infected mineworkers in South Africa.. AIDS Care.

[18] Adjei AA, Armah HB, Gbagbo F, Boamah I, Adu-Gyamfi C (2008). Seroprevalence of HHV-8, CMV, and EBV among the general population in Ghana, West Africa.. BMC Infect Dis.

[19] Boeckh M, Huang M, Ferrenberg J, Stevens-Ayers T, Stensland L (2004). Optimization of quantitative detection of cytomegalovirus DNA in plasma by real-time PCR.. J Clin Microbiol.

[20] Zuckerman RA, Lucchetti A, Whittington WL, Sanchez J, Coombs RW (2007). Herpes simplex virus (HSV) suppression with valacyclovir reduces rectal and blood plasma HIV-1 levels in HIV-1/HSV-2-seropositive men: a randomized, double-blind, placebo-controlled crossover trial.. J Infect Dis.

[21] Slyker JA, Lohman-Payne BL, Rowland-Jones SL, Otieno P, Maleche-Obimbo E (2009). The detection of cytomegalovirus DNA in maternal plasma is associated with mortality in HIV-1-infected women and their infants.. AIDS.

[22] Micol R, Buchy P, Guerrier G, Duong V, Ferradini L (2009). Prevalence, risk factors, and impact on outcome of cytomegalovirus replication in serum of Cambodian HIV-infected patients (2004–2007).. J Acquir Immune Defic Syndr.

[23] Kempen JH, Jabs DA, Wilson LA, Dunn JP, West SK (2003). Mortality risk for patients with cytomegalovirus retinitis and acquired immune deficiency syndrome.. Clin Infect Dis.

